# Perspectives of patients and physicians about neuroendocrine tumors. A qualitative study

**DOI:** 10.18632/oncotarget.24347

**Published:** 2018-01-31

**Authors:** Jordan Sibeoni, Wafaa Khannoussi, Emilie Manolios, Vinciane Rebours, Anne Revah-Levy, Philippe Ruszniewski

**Affiliations:** ^1^ Service Universitaire de Psychiatrie de l’Adolescent, Argenteuil Hospital Centre, Argenteuil, France; ^2^ ECSTRA Team, UMR-1153, Inserm, Paris Diderot University, Sorbonne Paris Cité, France; ^3^ Service de Gastroentérologie et Pancréatologie, Hôpital Beaujon, DHU UNITY, APHP, Clichy, France; ^4^ Inserm UMR1149, Université Paris–Diderot, Paris 7, France; ^5^ Service de Psychiatrie Adultes et du Sujet Agé, Unité Fonctionnelle de Psychologie et Psychiatrie de Liaison, AP-HP, Hôpitaux Universitaires Paris Ouest, Paris, France

**Keywords:** qualitative research, oncology, neuroendocrine tumors, gastrointestinal cancers

## Abstract

**Purpose:**

Gastrointestinal neuroendocrine tumors (NETs) are rare, complex to manage, and often have a chronic course. Qualitative methods are a tool of choice for focusing on patients' and physicians’ points of view especially when dealing with a complex and rare disease. Nonetheless, they remain undeveloped in research related to NETs. This study aimed to explore the experience of NETs among both patients and their physicians and to cross their perspectives for the purpose of finding pathways to improving care.

**Results:**

Our analysis found two themes: (1) the questions raised by this disease, and (2) the complex experience of this singular disease. Our findings underlined the experience of confusion found among patients regarding the patient's unusual somatic experience and around the question of vocabulary, i.e. the naming of the disease and the semantic field of severity in the medical discourse.

**Conclusion:**

Means for reducing the confusion that patients experience in this disease are needed. The explanations that the physician offers to the patient must clarify the issues related to NETs. We therefore propose a statement that all physicians can use to support patients diagnosed with neuroendocrine tumors to clear up potential confusion.

**Methods:**

We conducted a qualitative study, based on 40 semi-structured interviews, in a specialized department of gastro-pancreatology. Participants, purposively selected until data saturation, came from two different sub-samples: (i) patients with a metastatic NETs (*N* = 20) and (ii) their referring physicians (*N* = 10). The data were examined by thematic analysis.

## INTRODUCTION

In the field of gastrointestinal cancers, neuroendocrine tumors (NETs) stand out for their rarity, the difficulty of their diagnosis, their often better prognosis, and their complex and long management.

These tumors account for 1% of all gastrointestinal cancers, although their incidence appears to be rising [1, 2]. They can be nonfunctional and engender nonspecific symptoms or remain asymptomatic for a long period. Globally, prognosis is better than for the most frequent gastrointestinal cancers, with an overall 10-year survival rate of 46.5% [1], and the patients’ overall condition is also globally good, with a median Karnofsky performance status score of 90 [3]. Management is therefore often that of a chronic disease with a slow and uncertain course. NETs can be no progressive for long periods and then evolve to a more aggressive phase with a poor prognosis. Treatment is therefore complex, ranging from simple monitoring to surgery, liver-directed procedures and medical treatment including the use of somatostatin analogues, conventional chemotherapy, targeted therapies, or vectorized metabolic radiotherapy [4, 5].

Qualitative studies remain undeveloped in research related to NETs. To our knowledge, only two qualitative studies have been published [6, 7]. Their results underlined the diagnostic difficulties and the importance of care at a specialized center. No study has ever crossed the perspectives of patients and physicians on this subject. Using qualitative methods, this study aimed to explore the experience of NETs among both patients and their physicians and to cross their perspectives for the purpose of finding pathways to improving care.

## RESULTS

We conducted 40 interviews and included 20 patients and 10 physicians. On average, each physician participated in 2 interviews (range 1–4). The age range of these patients – 11 men and 9 women – was 53 to 75 years. They had been receiving care for their NET for from 1 to 17 years. Table 1 summarizes the patients’ characteristics and Table 2 the physicians’ characteristics. No patient was receiving psychological care.

**Table 1:** Patients’ characteristics

Patients	Sex	Age	Marital status	Date of discovery	Primary tumor site	Site of metastases	Comorbidities	Treatments received	Treatments underway	Referring physicians
P1	M	68	D	2010	Small intestines	liver peritoneum	0	cx CMT TC SRS	SRS	MD1
P2	M	53	M	2010	Small intestines	Liver, lymph nodes	Hypertension HCT	cx SRS	SRS	MD1
P3	M	72	M	2007	Small intestines	Liver, bones, lymph nodes, lung	0	cx TC SRS	SRS	MD2
P4	F	67	M	2012	Small intestines	liver lymph nodes	0	cx EHIA SRS	SRS	MD2
P5	F	70	M	2012	Pancreas	liver	Breast cancer	cx SRS	SRS	MD3
P6	M	64	M	2005	Small intestines	liver lymph nodes peritoneum	Hypertension	cx CMT SRS	SRS	MD4
P7	M	67	M	2005	Pancreas	liver		SRS targeted chemotherapies	none	MD1
P8	F	69	M	1999	Pancreas	liver		SRS chemoembol surg	chemoembol	MD5
P9	M	75	M	2013	Small intestines	liver peritoneum	adenoma prostate	SRS surg	SRS	MD6
P10	M	75	M	2004	Pancreas	liver suprarenal	Hypertension diabetes	SRS surg	SRS	MD2
P11	F	61	M	2011	Pancreas	liver lymph nodes	Diabetes	SRS surg chemoembol	chemoembol	MD3
P12	M	67	M	2003	Small intestines	liver		SRS surg	SRS	MD3
P13	F	71	M	2000	Pancreas	liver		Surg SRS targeted chemotherapies	SRS	MD1
P14	F	63	M	2013	Pancreas	liver	Hypertension	Targeted chemotherapies	targeted therapies	MD4
P15	M	74	M	2013	Duodenum	liver bones		Surg chemoembol chemo	chemo	MD5
P16	F	53	M	2010	Pancreas	liver		SRS chemoembol	chemoembol	MD6
P17	F	54	M	2011	Pancreas	liver		Surg targeted chemotherapies	chemo	MD7
P18	M	73	M	2011	Pancreas	liver	K prostate MI	none	none	MD8
P19	M	61	M	2015	Pancreas	liver		none	none	MD9
P20	F	70	V	2015	Pancreas	liver		SRS	SRS	MD10

NB : All the patients recruited chose to participate except two, one because of anxiety in talking, and the other for organizational reasons.

**Table 2:** Physicians’ characteristics

	Gender	Age	Medical specialty	Status
MD1	F	45	Gastroenterology	Attending physician
MD2	M	62	Gastroenterology	Professor, dean of faculty
MD3	F	40	Gastroenterology	Professor
MD4	M	62	Gastroenterology	Professor, head of department
MD5	F	46	Gastroenterology	Attending physician
MD6	M	58	Digestive oncology	Professor
MD7	F	33	Gastroenterology	Senior resident
MD8	F	31	Gastroenterology	Chief resident
MD9	F	36	Gastroenterology	Fellow
MD10	F	32	Gastroenterology	Chief resident

### Results of the thematic analysis

Our analysis found two themes that structured the experience shared by the patients and their physicians: (1) the questions raised by this disease, and (2) the complex experience of this singular disease. The direct quotations illustrating the results are presented in Table 3.

**Table 3:** Quotations

1. The questions raised by this disease 1.1 What is this disease?	
A sudden discovery	
Q1	P 10: I had symptoms pretty suddenly. I started vomiting and it had nothing to with a meal – that was obvious in view was what came up –, accompanied by intense diarrhea — like a stomach flu.
Q2	MD5: It was a woman I met 3 years ago. She had been referred to me because they had discovered, completely by chance, which happens pretty often, a neuroendocrine tumor of the pancreas, which already had hepatic metastases at the start.
A rare and elusive disease	
Q3	P1: So, for 5 years. It was discovered late because... I was being seen, being treated for diverticula... well it was ... a neuroendocrine cancer; so there was confusion at the start.
Q4	MD18: It was a kind of scientific curiosity, that was very interesting to physicians, but at the same time, not very dangerous […] They came from an environment where they were told, “oh, wow, you have a neuroendocrine tumor”… Already, you need a specialized department, because it is rare. So they have the impression that they really have a rare disease within a rare disease. That's what I call a curiosity.
Q5	MD12: I had him do this scintigraphy, which is ultrasensitive, where on we might see something more. In any case, we see things on almost a millimetric scale that you cannot see on more conventional imaging, such as scanners or MRI. And there, on this scintigraphy, there were spots, which were minimal, very little, on the liver.
**1.2 What is happening in the body ?**	
The body's silence vs aggression of the body	
Q6	P5: I had no symptom, didn't feel sick, which meant … I would say for 6 months, I was saying to myself: what's the point of this?
Q7	P5: Really, I don't think about it. I think about it, because, I come here, and then because I have to think about it to take my medication regularly. There is nonetheless medical care […] Now, I'm content to come. Because I say to myself: now, I'm going to know if this treatment is really effective, or if in the meantime, it's gotten worse, because something's happening inside, but I am not capable of realizing what's happening inside me.
Q8	P17: Professionally, I’ve adjusted. The handicaps I encounter, there, it's a little harder. I try to adjust anyway, but I admit that it's a little more complicated, because I see that it slows me down, it's painful.
Q9	P 14: My body, even the pancreas, it made bypasses, because it holds onto the veins and the arteries, the tumor, it holds on to them all, and my body has made bypasses, nodes so that the blood passes.
Q10	MD 16: It's what I call, with some patients, the desert of the Tartars. You have binoculars, you wait for the disease, nothing happens, it's the desert. You’re still worried, but you see nothing, and you’re waiting for there to be an army that arrives to face you.
Q11	MD 14: That's one of the tumors with a poor prognosis. It's a somewhat particular case. It's not the bog-standard endocrine tumor that, even when it's metastatic, the patient can be alive 15 or 20 years later. This one, it's a little more complicated […] I was pretty pessimistic at the beginning […] it was a pretty aggressive tumor, and I didn't think she'd still be alive today.
Imagining the damage to the body	
Q12	P5: Internally, I didn't realize, but externally, nothing happened, I had no special feelings of illness, I didn't feel bad anywhere…
Q13	P6: My representation of the disease, bah, it's the one the scanners make.
Q14	P12: The liver is going to regenerate all by itself, a little like that phoenix in Greek mythology.
Q15	MD 2: It's difficult to know how he represents these things to himself. I don't know if he really does.
**1.3 Is it a serious disease?**	
Q16	P9: For now, it's very good. On the other hand, they warned me that it could evolve, because it's a disease that's not like a standard cancer. It's a disease that's sort of unknown, and then besides each patient is different, I suppose. That makes it harder.
Q17	P11: I didn't have chemo, no radiology(…) so it's not cancer.
Q18	P9: Given that I don't really have any constraints, I try to live as normally as possible, as if nothing was going on. So much so that my wife seems to think I'm not being aware enough of what's happening to me.
Q19	P3: They tell me I'm a good patient… even very healthy.
Q20	P5: I don't consider myself sick, at all. […] It's true that I have the impression a little that it lives alongside me… [Laughs] certainly not in my everyday activities.
Q21	MD5: I say to myself: but what must they think, the people whom I am always trying to make understand, either explicitly or implicitly, that finally, they have a metastatic cancer but after all, it's not as serious as that. It's better than catching scarlet fever. I'm joking a little, but it is nonetheless pretty incredible.
**1 .4 A name for this disease?**	
Q22	P16: Go say that to Professor XXX; Tell him that I have cancer, you’ll see. He will tell you, “Ms. XXX does not have cancer, she has a neuroendocrine tumor.” It's not a cancer so therefore the metastases are not cancerous. Except when they grow, in my opinion, it's already a different story. In fact, it's treated like a cancer anyway.
Q23	MD 1: It's complicated. […] I talk about a malignant neuroendocrine tumor. So they say: “is it cancer?” I answer that “it's a malignant tumor, so in that sense, it's cancer, but if you call it cancer, it's a cancer that is very different from what we usually call cancer.”
Q24	MD 10: Cancer, no. No, because, for me, it's not at all the same… In the end, I find that it's too harsh for what it is. For me, it's not cancer in the common sense of the term.
**1.5 The position of the expert specialist: answers without questions**	
Q25	P18: It's clear that he is extremely competent, has great experience, all that. They told me that here, they’re at the top level for the pancreas… for diseases of the pancreas.
Q26	P6: I landed with Professor XXX who told me that it was still operable, because he had an excellent team… Which implied a little that here we are better than elsewhere. I had complete confidence in his hypothesis.
Q27	P18: I see him before the end of the year with a blood test, then six months later, with a scanner as well to verify, check that there are no metastases. But he told me that the tumors are not very large, so we monitor, and that's all. So I left, reassured.
Q28	P14: Yes, I trust them, totally. I have always trusted them totally, I let them guide me, and I'm not unhappy with the result..
Q29	MD 1: I think that she very rapidly felt that she had arrived somewhere where we knew her disease very well, and that this disease, there are a lot of places where they don't know it.
Q30	MD3: It's sometimes more my disease than theirs.
**2. Complex experience of a singular disease** **2.1 Distress**	
Q31	P6: Honestly, I have no reason to complain … they said that I was sick and that I was going to go to the hospital but I said to myself: that's it, they are going to take care of me. Now, I can take it easy. The problems stop at the hospital door.
Q32	P12: What worries me, the death of a person, you know, at my age, and in relation to my children, it's difficult. I'm afraid of… It's difficult for my wife and me, because we were a very tight-knit family.
Q33	P1: Fear… I don't plan any more, I don’t, I'm afraid to … I don't plan, it's finished, because there is always something that comes up with it and it's not possible …I’ve given up all my plans.
Q34	P10: I play the ostrich. What will knowing change? This is not what will make it resorb… The people, they are traumatized. They exaggerate a little. In any case, in my opinion, they take risks.
Q35	MD 18 : There's a dimension that we control less well, maybe, that's the psychological repercussions.
Q36	MD5: We are in a situation, I would say, of relative tranquility, of disease stability, where I'm in front of someone who is in exactly the same physical shape as me, maybe even better, I don't know. The question of death, it can come in in some patients at some point, but generally in people who have already had several lines of treatment, who feel the vise tightening around them.
Q37	MD15: No, I don't feel anxious. While, for example, when I saw him last time, it was to tell him that the treatment had worked well (…) he expressed more lassitude about having to continue than joy in saying that it was working.
Q38	MD13: She's a woman who is extremely positive in her approach to things, who always tends to try to minimize her disease, and who therefore is always very happy about all the therapeutic decisions we tell her about.
**2.2 Confusion**	
Q39	P2: I don't know anymore… Then they did a treatment with Somatuline that blocked my tumors. And progressively, as I come for treatment, some of them have shrunk. Now normally, they shouldn't have moved. And I don't have any more of them. I still have as many. They told me that they shouldn't have shrunk either. So, I don't know anymore.
Q40	P4: I tell myself: maybe your body is going to win the battle. In life, there's only battles [laughs]. I don't think I'm wrong.
Q41	P3: I don't understand anything [about this disease]. It's something that makes me sick, that's all, that might... I don't know, degenerate. I don't know…
Q42	P9: I don't understand the disease, but ok, it's not serious. What's important, is that it leave me in peace. Too bad.
Q43	P10: What I did was perhaps experienced as provocative, but in fact, it was for the pedagogues, to get it into their heads that the patient is not necessarily a medical student, that it has to be explained in words the patient understands. It is necessary to explain in clear terms, even though these are things… even more if these are complicated things. In a word, put yourself within the patient's reach, don't talk like all technicians, not only in medicine, who have their own jargon, so that if you’re not initiated to their technical…
Q44	MD10: It's true that he doesn't talk much either. […] There, I understood that he hadn't understood where the lymph node was. I explained that we did a scintigraphy to see if there weren't any others. I think, and I hope, that he understands that they are nodules related to his gastrinome, that is, with the pancreatic tumor he had in 2004.
Q45	MD6: He had not really understood the association between the primary tumor and the secondary lesions, although intellectually, he is among the most educated people in my practice. One day, he surprised me a little in making a comment that proved that basically he really had not understood his disease at all.

### The questions raised by this disease

The patients and doctors shared the same questions about these metastatic neuroendocrine tumors.

#### What is this disease?

#### A sudden discovery

Most patients reported that their disease was discovered suddenly, most often “by chance”, without any signs, and without any characteristic symptoms. Some patients described a fortuitous discovery, while others described vague signs such as fatigue, stomach pain, or “a common backache.” Many patients also described vomiting and diarrhea, symptoms that did not alarm them or alert their general practitioner to something serious (Q1).

The physicians interviewed also mentioned this sudden, accidental discovery of the disease (Q2).

#### A rare disease

Most patients described their disease as rare and little known even by healthcare professionals. Most also reported diagnostic errors leading to diagnostic delay along their health care pathway (Q3)

The physicians interviewed also underlined the rarity of this disease; some even described it as “a scientific curiosity” (Q4). They insisted especially on its elusive nature: a NET is a disease that can be fully grasped only by imaging procedures (Q5).

#### What is happening in the body?

Patients and doctors both found it difficult to express what is going on in a body with a NET.

#### The body's silence versus aggression of the body

Subsequent regular medical follow-up became the proof and the only sign of their disease (Q6). The symptoms associated with the NET itself were described as “not much” or “not annoying” (Q7). Although most patients stressed the absence of somatic distress, some complained about a negative physical effect in their daily lives: fatigue, weakened immune system, or insomnia. The term disability was sometimes used (Q8). Still others experienced these physical changes as an aggression and a degradation of their self-image (Q9).

Similarly, all the physicians mentioned the absence of physical signs on a daily basis, to the point that one doctor stated that he “treated scanners.” Nonetheless, they did not think that the absence of signs meant any absence of danger, and their discourse could have introduced the idea of waiting for something worse (Q10). The doctors also talked about “aggressive disease” and its sometimes painful consequences patients (Q11).

#### Imagining the damage in the body

Many patients found it hard to imagine or describe what happens inside their bodies (Q12). Some relied on imaging examinations (Q13) or on their knowledge about some organs (Q14). Similarly, the doctors were at ease using anatomical vocabulary to help their patients understand their disease. On the other hand, they found hard to describe their patients’ mental representations (Q15).

#### A serious disease?

Most of the patients considered that they did not have a serious disease, even though they used the words “cancer”, “tumors”, and “metastases”. Following the doctors’ discourse, they used the terms “chronic disease” or “slow course” with the idea that the disease might evolve to a more serious phase (Q16). Others judged this seriousness according to the treatment they received (Q17).

Many patients explained that they did not suffer and were not “bothered” by their disease (Q18), that they live the paradox of being “healthy patients” (Q19). Some even considered that the disease was a thing apart, detached from their real life (Q20).

The physicians did not qualify the NET as a serious disease but nonetheless wondered what the patients felt when they heard the term metastatic cancer associated with the idea that it is not a serious disease (Q21). They were also aware that their patients experienced the paradox of being healthy patients, which they attributed to the lack of perceived symptoms.

#### A name for this disease?

All the patients raised the question of the terminology used to talk about their disease and their perplexity about the terms the physicians used during their medical follow-up to name this disease. Many patients considered their disease to be “equivalent to cancer” and thought that their doctor had not pronounced the word cancer because he or she considered it a taboo (Q22).

Moreover, we found some 30 different expressions used by the patients to name their disease: lazy cancer; carcinoid; this glucagonoma; this thing; pimples or spots on the liver; little dots; funny critter; bloody trick; slowly progressive disease; nice disease; insidious disease, sarcophagus, vampire, a cousin of the cancer family. The term used most often by patients was the word “truc”, which translates literally as thing, thingie, and even trick; it thus perfectly expresses their difficulty in naming what they have.

Some physicians said they used the term cancer to relativize it (Q23). Other refused to use this word, while recognizing that their patients used this word – questioning it, pronouncing it, and finding it a source of perplexity (Q24).

#### The position of the expert specialist: answers without questions

The dimension of the “expert specialist” was often a key detail in the patients’ discourse (Q25), mentioning ultra-competent and trustworthy physician (Q26) holders of knowledge and of all the answers and reassuring follow-up (Q27, Q28).

The physicians were aware that their status as an expert provided their patients with reassurance but might lead to some inhibition on the part of the patients (Q29). Some physicians even mentioned the sensation that the NET was more the physician's disease than the patient's (Q30).

### Complex experience of a singular disease

#### Distress

On the one hand, some patients relativized the disease and considered that they had no reason to complain (Q31).

On the other hand, they evoked very explicitly their anguish about death. Terms in the lexical field of fear were recurrent in patients’ discourse (Q32). Some also mentioned that they have found it impossible to plan for the future since their disease began (Q33). Many said that they avoided talking about their disease and explained that they did not want more specific information about it (Q34).

The physicians found it difficult to identify the psychological effects of the disease on their patients (Q35). They mentioned the patients’ anxieties about death, but tended to relativize and minimize them (Q36). They sometimes observed the patients’ distress but did not succeed in understanding its origin or attributed it to causes exterior to the disease (e.g. family problems, mourning, depressive condition before the diagnosis).

Nonetheless, the physicians reported awareness of the emotional experience associated with treatments and to the weariness expressed about their constraints and side effects (Q37). They also identified defense mechanisms in their patients, in particular, avoidance and minimization (Q38).

#### Confusion

Generally, patients often seemed lost in the face of this disease, unable to make sense of their experience of either the disease or their care (Q39). Most often, their discourse about their experience appeared vague and confused. Raising the question of their experience led to the disorganization of some patients’ discourse (Q40). Moreover, very few patients understood their disease despite medical follow-up that had often gone on for a very long time. This confusion or lack of understanding was clearly expressed in the interviews (Q41). Many patients explained that they had used the internet to try to understand their disease. Astonishingly, most of the patients appeared to tolerate, even integrate, this incomprehension (Q42). Finally, several patients reported the impression that the physician was hiding some information about the disease severity while others had the feeling that they and the doctor did not even speak the same language (Q43).

The physicians recognized this lack of understanding but related it to a lack of objective knowledge about the disease (Q44). Some physicians reported moments of confusion and incomprehension in their patients, which perplexed them (Q45).

## DISCUSSION

This is the first study reporting a structured account of the experience of NET by patients and their doctors.

The patients’ narratives contain a dimension of emotional distress, simultaneously anxiety about the future and about the impact of NET on their daily life. These results confirm those already reported in the literature. Several studies have mentioned impairment of the health-related quality of life (QoL) in patients with NETs [8, 9]. A study of QoL in such patients, also found impaired emotional health in most respondents, in particular, a high anxiety level and worry about the uncertainty of their future [10]. Several specific treatment interventions have been proposed to improve the QoL of these patients, including patient education programs [11]; none of these interventions has thus far concerned physician-patient communication.

In our study, physicians perceived their patients’ distress but did not attribute it directly to their NET experience. It appears in our study that the patients’ distress may be associated to their experience of confusion and to difficulties in communication between the physician and the patient.

### The question of confusion

The primary result of this study is the important experience of confusion found among patients. The International Neuroendocrine Cancer Alliance study reported that 39% of the patients questioned were confused about the management of their disease [10]. In our study, we had access to the details of this experience of confusion, which appeared to have two aspects – somatic and semantic.

A first confusing aspect concerned the patient's unusual somatic experience: a non-specific clinical picture, difficulty in imagining and localizing the damage within the body, and especially a non-symptomatic experience, the body's silence, which creates the impression of not being sick.

The second confusing aspect concerns the naming of the disease and the semantic field of medical discourse. If we follow the WHO classification, which reserves the term carcinoma for poorly differentiated tumors, NETs are not cancers in the strict sense of the word [12]. Nonetheless, most of the patients and physicians in our study explicitly used the term cancer. The distinctions between tumors and cancer are difficult for patients to understand. The 30-odd expressions found in these accounts to name NETs demonstrate the extent to which it was difficult to share a common designation.

Moreover, the question of vocabulary also played a role in the confusion about the severity of the disease; on the one hand, physicians tell patients that it is a slowly progressive disease, not necessarily serious; on the other hand, they inevitably use the words metastasis, tumor, and cancer. The vocabulary surrounding NETs is undeniably that of cancer and in everyday language, when patients hear the words “metastatic cancer”, they understand them as designating a serious disease with a substantial risk of death. Here, not only is the term metastasis not associated with the idea of severity, but it also does not necessarily involve treatment procedures; instead it is sometimes managed by monitoring. This “watch and wait” position probably contributes to the patients’ incomprehension and anxiety.

The medical explanations about this disease and the specialists’ efforts to reassure patients do not succeed in reducing the patient's confusion and may sometimes preserve or even nourish it. The study by Feinberg *et al*. has already pointed out how hard it is for patients with NETS to obtain understandable and appropriate (meaningful) information from their family physicians [5]. The different context of our study – in an expert center – suggests that the patient's incomprehension is linked more to this experience of confusion than to a lack of information and objective data about the disease.

### Perspectives on treatment and training

In clinical practice, it appears necessary to find means of reducing the confusion that patients experience in this disease.

The explanations that the physician offers to the patient must clarify the issues related to NETS. We propose a statement that all physicians can use to support patients diagnosed with NETS:

*“*You have been diagnosed with a particular type of cancer, called a neuroendocrine tumor. This tumor developed in your (pancreas, small bowel, …) and metastasized in your (liver, lung…..) It is a rarer cancer than those we usually talk about, such as cancer of the pancreas or colon. More rare does not mean more serious, however. In fact the contrary is true here. Many patients have no or only a few symptoms. Moreover, neuroendocrine tumors develop much more slowly than other cancers and can even fail to grow at all for fairly long periods (several months, or even years). There are a number of effective treatments available. They have variable side effects. The decision to start a treatment depends on the risk that the disease causes you. If the disease is not threatening (small tumors that are not growing), we can just monitor it and only start treatment if it changes. In the other cases, you will be offered a treatment. It will be chosen according to the extent and importance of the disease.*”*

This proposed statement seeks to clear up potential confusion, especially semantic confusion as it explicitly used the term cancer. It meets patients’ needs (i.e. silent symptomatology, name, evolution, treatment and monitoring) including the need to improve patient-physician communication.

It can be used in specialized medical consultations but also to support the training of physicians who may care for patients with a NET (e.g., oncologists and gastroenterologists). To validate its use, we are testing this proposed statement in the department of gastro-pancreatology at Beaujon Hospital but also in two other medical oncology departments.

A recent meta-analysis examined the issue of announcing bad news in oncology and concluded that doctors announcing cancer diagnoses require specific training [13]. We propose to extend this work and this training to the announcement of complex rare diseases such as NETs.

### Limitations

Interpretation of this study requires consideration of its limitations. First, it took place in France and some of our results deal directly with linguistic and terminological aspects specific to French; caution is thus needed in transposing them to other care settings. Second, all the participants in this study were recruited from a hospital that is an expert reference center for this disease. Our results therefore exemplify this situation but cannot account for other medical settings, such as for example that of general medicine, where the aspects of diagnostic delay remains important [5]. Finally, this was a single-center study; it would be interesting to see the results of other studies reproducing this design in other medical settings. Given the specific context of this study and its qualitative methodology, we would not claim that it is generalizable; instead, we think that it meets the aim of qualitative research: it may be transferable to other contexts.

## METHODS

Our exploratory single-center study took place in the department of gastro-pancreatology at Beaujon Hospital, specialized in the management of NETs. Paris-Descartes University review board (CERES) gave the ethical approval (IRB number: 20140600001072).

### Participants and recruitment

Our sample was constituted by two different subsamples: (i) patients with a metastatic gastrointestinal NET and (ii) their physicians. The doctors could be interviewed more than once (once per patient included). In total, 40 interviews were conducted. Inclusion criteria for patients are reported in Table 4.

**Table 4:** Inclusion and exclusion criteria

Inclusion criteria	Exclusion criteria
Age: 18 or older	Age < 18
Treatment or monitoring started at least one year before the interview	Psychiatric disorders or impairments of cognitive function that would prevent a useful interview
For a metastatic gastrointestinal NET, grade 1 or 2 stable at inclusion NET diagnosis criteria: Confirmed either by biopsy of the primary tumor or liver metastasis And by immunohistochemical expression of both chromogranin A and synaptophysin Assessment of tumor differentiation according to the WHO 2010 classification and its proliferation rate was evaluated by estimates of Ki67 Tumors with Ki67 ≤ 2% = grade 1 Tumors with Ki67 3–20% = grade 2	
Referring physician agrees to participate to the research	

The clinician coordinator (WK) presented the study design and objective to the referring physicians and identified with them patients who met inclusion criteria. She asked patients who met the inclusion criteria (and physicians) to participate in this study and scheduled the interview the day of the next appointment (or at the first available day). To ensure an in-depth exploration, we adopted a minimum variation purposive sampling strategy [14], that is, we limited our sample to a level of severity and to day hospital care to obtain a homogeneous sample that would enable us to explore our research question in depth [15]. Inclusion continued until saturation was reached (i.e., subsequent interviews provided no new elements), according to the standards of qualitative research [16, 17].

### Data collection

We used semi-structured interviews conducted by experienced qualitative researchers (WK, JS, and EM following a topic guide composed by open questions -Table 5). The interviews took place in a private room in the hospital in which the patients were treated, and were audiotaped and transcribed verbatim. The same researcher interviewed the patient and the referring specialist. The interviews have been anonymized.

**Table 5:** Exploration areas

Exploration area n°1: History of the disease (chronology of events, the announcement of the diagnosis, etc.)
Exploration area n°2: Experience of the disease (physical experience, emotions, repercussions in daily life, role of family members and close friends, etc.)
Exploration area n°3: Experience of care (the different treatments, monitoring, and follow-up)

### Data analysis

We performed a thematic analysis [18]. First, we coded the material using descriptive codes. Then conceptual notes were drafted, through processes of condensation, comparison, and abstracting the initial notes. Connections with notes were then mapped and synthesized, and emergent themes developed. The interview of a given patient and his/her physician were analyzed together, in order to compare and contrast both perspectives. Table 6 summarizes the different stages of our thematic analysis. The analyses were independently performed by 3 researchers (JS, EM, ARL) using Nvivo 11, and their subsequent triangulation ensured the rigor and intersubjectivity of the analytic process. Discrepancies were negotiated within the research team during regular meetings until agreement was reached. Reporting followed the COREQ statement [19].

**Table 6:** Inductive thematic analysis

	Activities	Rationale
Stage1	Repeatedly read each transcript, as a whole.	Obtain a global picture of the interview and become familiar with the interviewee's verbal style and vocabulary. Each new reading of the transcript might also provide new perspectives.
Stage2	Code the transcript by making notes corresponding to the fundamental units of meanings.	Make descriptive notes using the participant's own words. Pay particular attention to linguistic details, such as the use of metaphor.
Stage3	Make conceptual notes through processes of condensation, abstraction, and comparison of the initial notes.	Categorize initial notes and reach a higher level of abstraction.
Stage4	Identify initial themes. Provide text quotes that illustrate the main ideas of each theme.	Themes are labels that summarize the essence of a number of related conceptual notes. They are used to capture the experience of the phenomenon under study.
Stage5	Identify recurrent themes across transcripts and produce a coherent ordered table of the themes and sub-themes.	Move from the particular to the shared across multiple experiences. Recurrent themes reflect a shared understanding of the phenomena among all participants. During this more analytic stage, researchers try to make sense of the associations between the themes found.
